# Internet-based interventions for perinatal depression and anxiety symptoms: an ethnographic qualitative study exploring the views and opinions of midwives in Switzerland

**DOI:** 10.1186/s12875-022-01779-8

**Published:** 2022-07-14

**Authors:** Josephine Beerli, Ulrike Ehlert, Rita T. Amiel Castro

**Affiliations:** Institute of Psychology, Department of Clinical Psychology and Psychotherapy, University of Zurich, Zürich, Switzerland

**Keywords:** Perinatal mental health, Internet-based interventions, Midwives, Perinatal depressive symptoms, Perinatal anxiety symptoms

## Abstract

**Background:**

Mental disorders such as depression and anxiety are common during pregnancy and postpartum, but are frequently underdiagnosed and untreated. In the last decades, internet-based interventions have emerged as a treatment alternative showing similar effectiveness to face-to-face psychotherapy. We aimed to explore midwives’ perceptions of the acceptability of internet-based interventions for the treatment of perinatal depression and anxiety symptoms.

**Methods:**

In this ethnographic qualitative study, semi-structured interviews were conducted with 30 midwives. We followed the Consolidated Criteria for Reporting Qualitative Research Checklist (COREQ). Audio-recorded interviews were transcribed verbatim and analysed using framework analysis. The identified framework categories were rated individually by two independent raters. Krippendorff’s alpha coefficient was used to ensure the reliability of the rating.

**Results:**

Four main themes emerged: midwives’ experience with patients’ mental health issues; the role of healthcare workers in women’s utilisation of internet-based interventions in the perinatal period; the overall perception of internet-based interventions; and recommendation of internet-based interventions to perinatal women. Twenty-five of the 30 participants viewed internet-based interventions as an acceptable type of intervention, which they would recommend to a subgroup of patients (e.g. women who are well-educated or younger). All except for two midwives identified themselves and medical doctors as key figures regarding patients’ utilisation of internet-based interventions, although a third of the interviewees highlighted that they needed sufficient information about such interventions. Finally, several participants suggested features which could be relevant to develop more acceptable and feasible internet-based interventions in the future.

**Discussion:**

Participants’ overall perception of internet-based interventions for perinatal depression and anxiety symptoms was positive. This study underlines the importance of considering midwives’ views about internet-based interventions for perinatal mental health care. Our findings have implications for the practice not only of midwives but also of other maternity care professionals. Future studies examining the views of other health professionals are warranted.

## Highlights

• There is a lack of studies on health professionals’ views about women’s utilisation of internet-based interventions in the perinatal period.

• Our findings suggest that midwives have a positive perception of internet-based interventions, despite identifying disadvantages and having concerns about their use.

• These findings are encouraging and contribute to the continued efforts to develop internet-based mental health interventions as a way to support perinatal women screened or diagnosed with anxiety and/or depressive symptoms.

## Background

Pregnancy and the postpartum period pose an increased risk of mental disorders such as anxiety or depression [1, 2]. Poor mental health during the perinatal period – defined as disorders commonly presenting in pregnancy and up to 1 year after birth [3] – has often been associated with adverse effects in mothers [4] and babies [5, 6]. Even though other studies report no evidence of a relationship between maternal postpartum depression and child physical and neurodevelopmental problems [7], perinatal mental health remains central to maternal and child outcomes.

Treatment rates for perinatal mental illnesses are low [8, 9] and various treatment barriers have been identified, including stigma [10, 11], time scheduling [8] and lack of information [10]. Internet-based interventions can redress some of these barriers, and have shown similar effectiveness to face-to-face psychotherapy [12, 13]. Evidence suggests that in cases of mild to moderate perinatal depression and anxiety symptoms, internet-based interventions may effectively and significantly reduce symptoms [14–17]. To date, the majority of studies have focused on postnatal depressive and anxiety symptoms [14, 16, 18–22], while fewer have assessed the effectiveness for antenatal symptoms [17, 23]. With regard to the latter, one study found lower levels of depressive symptoms following internet-based cognitive behavioural therapy (CBT) compared to a treatment as usual group, with large effect sizes [17]. Another study reported a large reduction in anxiety symptoms and a moderate improvement in depressive symptoms 9 months post intervention [23]. In a randomized controlled trial, Milgrom and colleagues recently examined the efficacy of an internet-based intervention compared to face-to-face cognitive behavioural treatment in women with diagnosed postpartum depression. The authors concluded that the online intervention was as effective as face-to-face treatment in achieving remission from a diagnosed postpartum depression, and superior in encouraging and maintaining a reduction in symptom severity over a 21-week follow-up [24].

Available studies using internet-based CBT and behavioural activation have also demonstrated significant short- and long-term reductions in perinatal depression and anxiety symptoms, including women in a clinically severe symptom range [25–27]. For instance, O’Mahen and colleagues reported lower postpartum depressive symptoms after a 12-week behavioural activation therapy [27], and Milgrom et al. found a 4-fold increase in remission in women diagnosed with postpartum depression (assessment based on the Diagnostic and Statistical Manual of Mental Disorders) 3 months after enrolment in a CBT online intervention [14]. Finally, according to systematic analyses, internet-based interventions show medium-sized effects in reducing perinatal depression or anxiety symptoms [28–30], and improvements in perinatal depressive symptoms are maintained at both short- and long-term follow-ups [28, 31]. Perinatal depression and anxiety are highly comorbid [32] and share similar aetiology and maintenance processes [33]. Despite this, few online interventions have targeted both depression and anxiety symptoms in perinatal women. Initial results suggest that internet-based CBT significantly reduces co-occuring depression and anxiety symptoms both in pregnant [20] and postpartum women [15].

Before investigating the application of internet-based interventions, it is necessary to consider their limitations. Firstly, according to the National Institute for Health and Care Excellence (NICE) guidelines, internet-based interventions during the perinatal period should be limited to women with mild to moderate depressive or anxiety symptoms [34]. Security issues such as personal data protection and data storage also constitute a challenge, particularly when using smartphones [35]. In addition, the impact of lack of personal contact and consequently women’s inability to benefit from the therapeutic alliance [36–38], may restrict effectiveness of internet-based interventions [36, 39]. Moreover, a large study capturing a wider demographic of women including those with reduced access to the internet and low technology literacy is lacking. Lastly, little is known about the rates of dropout from internet-based interventions for perinatal depression and anxiety symptoms, with reported rates ranging widely from 4.5–86% [40, 41], and lack of information on factors determining dropouts and non-responses [35].

Here, we will use the term *internet-based interventions* to refer to CBT interventions delivered over the internet. CBT is a widely used psychological intervention aiming to help patients adopt adequate coping strategies, with demonstrated efficacy in the treatment of anxiety and depression. Most internet-based interventions are accessed through a personal login [16, 17, 21, 42] and consist of three to twelve sessions [17, 20], usually completed in consecutive order [18, 19, 42]. Sessions generally include therapeutic components such as psychoeducation, goal-setting, coping with automatic negative thoughts, mother-baby bonding techniques, relaxation methods [14], homework assignments, and psychological assessment completed directly on the computer [35]. In the majority of internet-based interventions, patients can set their own pace in completing and working on the sessions [19, 21]. Internet-based guided interventions additionally offer individual support via email [17, 21] or telephone calls with coaches or therapists [16, 18], with some also offering peer-based forums [19, 42].

To date, research has mostly focused on the acceptability [43], feasibility [18, 44] and effectiveness [27, 41] of internet-based interventions in reducing perinatal depression and anxiety. However, little is known about the views and opinions of healthcare providers with respect to the acceptability of such interventions for women with perinatal depression and anxiety symptoms. This is highly relevant given that healthcare workers such as midwives, health visitors and medical doctors might influence women’s utilisation of internet-based interventions [45] by informing them about available treatments [46]. In Switzerland, midwives work closely with parents, providing support, counselling, and care from conception to the postpartum period [30, 47, 48]. Midwifery services are easily available, offered as a part of routine perinatal care, and covered by compulsory health insurance [49, 50]. Since 2017, pregnant women have been able to choose between midwife-led or physician-led maternity care [50]. The compulsory health insurance also covers up to 16 postpartum home visits conducted by midwives during the first 2 months postpartum [49]. Accordingly, in Switzerland, midwives are among the main professionals caring for women in the perinatal period and develop a relationship of trust with their patients due to their regular contact [51]. They qualify as expert opinion leaders and might play an important role in women’s decisions regarding specific treatments for perinatal mental illnesses [52].

During the perinatal period, midwives help to identify and support women in psychological distress [53]. Outpatient mental health care is widely available and easily accessible in the Swiss healthcare system, and general psychiatric care is commonly offered in inpatient obstetric units. Recent evidence from a Swiss study examining the prevalence of perinatal mental disorders and mental healthcare use among this population suggests that medication is the most frequent treatment provided for perinatal mental disorders, followed by medical or psychological care [54]. The Federation of Swiss Psychologists (FSP) [55] provides guidelines on the provision of psychological interventions using online technologies that meet adequate ethical standards. This is similar to the respective psychology or psychotherapy societies in countries such as the US [56], UK [57], and Australia [58], which provide guidelines on policy principles, professional competence, patient privacy and data protection [55–58] for the use of internet-based interventions.

Despite consistent evidence on the effectiveness of internet-based interventions, there is a lack of studies on health professionals’ views about women’s utilisation of internet-based interventions during pregnancy and postpartum [59]. Therefore, we aimed to explore midwives’ views and opinions on the acceptability of internet-based interventions for the treatment of perinatal depression and anxiety symptoms.

## Methods

### Sample

This is a cross-sectional qualitative study using a purposive sample of midwives (*N* = 30) from Switzerland and Liechtenstein. Eligible participants were midwives who were currently self-employed or employed in hospitals or birth centres, were fluent in German, aged 18 years or older, and had completed a Bachelor’s degree or a federally recognised vocational training in midwifery.

This study protocol was in accordance with the Declaration of Helsinki and ethical approval was obtained from the Ethics Committee of the Faculty of Arts and Social Sciences of the University of Zurich (Approval Nr. 18.10.6).

### Procedure

Our recruitment strategy entailed searching national midwifery registries, contacting the Swiss midwifery association, hospitals, birthing centres, midwives’ practices, and physicians’ practices, and then getting in touch with listed midwives. Participants were also recruited via social media (e.g. Facebook). Following contact with the study team, potentially eligible participants received a study information sheet and confirmed their eligibility. Recruitment took place continuously. From the 34 midwives who replied to the study team and received the study materials, four decided not to participate, citing privacy concerns. This resulted in 30 interviews, which were analysed in full and included in this study. Our decision to include all eligible participants was due to evidence suggesting that data saturation cannot be reliably determined [60] and may have a weak theoretical statistical foundation [61]. Participants who fulfilled the inclusion criteria and were interested in participating provided consent before commencement of the study. Prior to the interview, participants read a short text about internet-based interventions for depressive and anxiety disorders. The text defined CBT and its use in the online context, differentiated between guided and unguided internet-based CBT, outlined the advantages and disadvantages of this method, and presented evidence on internet-based treatment outcomes. Moreover, the text contained information regarding the NICE clinical guidelines recommendation that internet-based CBT should be available for women with mild to moderate depression or anxiety during the perinatal period (see Table 1). The interviews were conducted from July 2019 to January 2020, either face-to-face or via telephone by one of the authors (JB), who has a Master’s degree in clinical psychology and has previous research interview experience. Participants agreed to the audio-recording of the interviews.

**Table 1:** Brief information given to participants about internet-based interventions (adapted and translated from German)

• Cognitive behavioural therapy (CBT) has long been shown to be effective in reducing symptoms of depression and anxiety disorders. It explores the links between thoughts, emotions and behaviour, being a time-limited, structured approach used to treat several mental health disorders.	
• CBT aims to help patients to understand their ways of thinking and behaving, and provides patients with the resources to alter their maladaptive cognitive and behavioural patterns. As a consequence, it alleviates patients’ distress. Due to its structured nature, CBT is the most widely researched and empirically supported psychotherapeutic method.	
• Driven by advances in technology, for some years, there has been internet-based cognitive behavioural therapy (iCBT). iCBT is delivered through an online platform where patients log in regularly to a secure website, have access to online materials usually organized in 6–12 modules and go through the treatment protocol via audio and video lessons.	
• iCBT can be delivered in sessions/themes comparable to face-to-face psychotherapy sessions. Patients can also receive homework assignments to be completed before the next module and fill out computer-administered questionnaires assessing their symptoms. iCBT programs are particularly easy to access, and patients are able to determine the pace at which they wish to complete them, which can improve learning and retention.	
• Numerous studies have shown that particularly for depression and anxiety disorders, iCBT is effective in reducing symptoms in the general adult population and also in the postpartum population. The effects seem to be similar to face-to-face cognitive behavioural treatment.	
• Improved effects have been observed especially in guided iCBT (e.g. psychotherapist/clinician support or coaching) for anxiety and depression. In a guided intervention, the patient is supported by a psychotherapist or coach when working through the online treatment protocol. The support given should be facilitative in nature. Interaction between patient and therapist can take place by telephone, email or any other communication method.	
• In an unguided intervention, feedback is provided by the computer based on the patient’s achievements and there is no contact, personal or online, involved.	
• Common disadvantages associated with both guided and unguided iCBT include: data security issues, high withdrawal rate, not applicable for all patients, failure to detect problems in the therapeutic process.	
• The National Institute for Health and Care Excellence (NICE) guidelines actively suggest iCBT for women with mild to moderate depression or anxiety during the perinatal period. According to the NICE, iCBT could improve low treatment rates and increase privacy and anonymity while reducing stigma.	

### Materials

A semi-structured interview was developed by our research group based on previously published literature on the topic [59, 62] (see Table 2). It included six open-ended questions about six different topics as well as questions about participants’ education and work experience. However, if an answer was not fully comprehended, the interviewer asked follow-up questions. The interview was initially tested in the field [63]. Six midwives participated in the pilot phase, which examined the comprehensibility, clarity and acceptance of the interview questions. The interview was well accepted and understood by the pilot participants and no changes in content or wording were required. Therefore, data from the pilot interviews were included in the results. The interview guide was not changed at any point. Sociodemographic data such as gender, age, nationality, country of residence, educational level, percentage level of employment, marital status and annual income were also obtained from those who wished to provide this information.

**Table 2:** Topics of interest and main questions in the semi-structured interview

Topics of interest	Main questions from the interview guide
*Education and work experience*	What kind of education or training do you have?
	When did you finish your education as a midwife?
	Which midwifery training did you attend?
	How long have you been working as a midwife?
	Where are you currently working?
	How long have you been working in this practice/clinic/hospital?
	In your years working as a midwife, have you had any experience with women suffering from postnatal or prenatal mental illnesses?
*Usefulness of internet-based interventions*	Now that you have read the introductory text and have some background information on internet-based interventions, how useful do you think these interventions are?
*Utilisation of internet-based interventions*	Do you think that women who are suffering from perinatal mental disorders such as anxiety or depression would use an internet-based intervention?
*Advantages*	Imagine that one of your patients is using an internet-based intervention on a regular basis. What advantages regarding the use of this intervention method could you think of?
*Personal contact*	Regardless of whether it is face-to-face therapy or internet-based interventions, could you please describe how you perceive the relevance of the personal contact between therapist and patient?
*Disadvantages and concerns*	Imagine that one of your patients is using an internet-based intervention on a regular basis. Would you have any concerns? If yes, which one(s)?
*Comments*	Are there any remarks or experiences you want to share with us?

The research process was guided by an ethnographic approach, which aims to explore and examine cultures and societies [64]. This qualitative approach seemed suitable to explore the views and opinions of midwives, given that midwives can be seen as an individual group. The interview should feel like a conversation while providing insight into and an understanding of subjects’ views [64, 65]. Moreover, the ethnographic approach is sensitive to socio-cultural environments and to the human sense-making, beliefs and behaviours [65] which we aimed to explore here.

### Statistical analysis

Data analysis was conducted using Quirkos software version 2.4.1 [66] for qualitative data analysis. We followed the *Consolidated Criteria for Reporting Qualitative Research Checklist* (COREQ) [67] as it promotes transparent and complete reporting of qualitative methods. Participants did not have the opportunity to review, comment on or change their answers after the interview had taken place. Our main analytical approach was based on framework analysis [68], which has been consistently recommended for policy and healthcare research [69].

First, the audio-recorded interviews were de-identified and transcribed verbatim. Next, we systematically identified and organised the data into patterns and themes, following the five stages of systematization recommended by Ritchie and Spencer [68]. In the first stage, we listened to the interview recordings, read the transcripts thoroughly and identified relevant patterns related to the research questions. This resulted in 68 initial codes of interest obtained through open coding (e.g. scepticism about internet-based interventions, stigma associated with perinatal depression and anxiety symptoms). In the second stage, we grouped the codes dealing with similar topics into 10 broader categories through axial coding, developing a framework which incorporated our research aim. In the third stage, we reviewed and indexed the interview transcript excerpts to the previously developed categories (advantages, disadvantages and concerns, implementation, recommendation to women in the perinatal period, overall view, target group, midwifery education, skills and experiences, role of healthcare workers, digital era, and suggestions). All of the created codes and categories were discussed in our research group until agreement was reached. Ambiguous extracts fitting into more than one category were added to different framework categories. To enhance the reliability and rigor of the analysis and interpretation, the 10 categories were rated individually by two independent raters, one of whom was an educational researcher experienced in qualitative research and the other a psychology researcher experienced in internet-based interventions for perinatal disorders. The raters were asked to match the interview excerpts to the 10 framework categories developed in the second stage of analysis. Krippendorff’s alpha coefficient was used to ensure reliability of rating. In the fourth stage, the data were organised into chart form through the creation of an Excel file. Participants were placed on the y-axis while the 10 identified categories were variables placed on the x-axis. This allowed us to summarise the interviews while facilitating within- and between-participants analyses [70]. In the fifth and final stage of framework analysis, we mapped and interpreted the dataset as a whole, identifying themes and subthemes within the broader categories in order to explore midwives’ views and opinions on the acceptability of internet-based interventions for the treatment of perinatal depression and anxiety.

The identified themes were as follows: midwives’ experience with patients with perinatal mental health issues, role of healthcare workers in perinatal women’s utilisation of internet-based interventions in the perinatal period, overall perception of internet-based interventions, recommending internet-based interventions to women in the perinatal period. To gain further insight into possible factors influencing study participants’ views and opinions, group differences based on sociodemographic characteristics were computed with Kruskal-Wallis and Dunn-Bonferroni tests or Mann-Whitney U tests, where applicable [71–73].

## Results

Sociodemographic characteristics of the sample are presented in Table 3. All interviewees were female and aged between 28 and 57 years (M = 43.3, SD = 8.3). Participants’ work experience ranged from 4 to 36 years (M = 16.2, SD = 8.7) and the majority (*N* = 22) had completed a university degree. Sixteen participants worked part-time while twelve worked full-time. Almost all participants (*N* = 28) lived in Switzerland. The interviews lasted between 15 and 76 minutes (M = 30.71, SD = 11.90). Krippendorff’s alpha coefficient between raters was satisfactory (α = .75) [74].

**Table 3:** Sociodemographic characteristics of the sample (*N* = 30)

Variables	Value
**Age, mean (SD)**^a^	43.3 (8.3)
**Marital Status, n (%)**^b^	
Single	11 (39.3)
Married/Registered partnership	16 (57.1)
Divorced/Annulled registered partnership	1 (3.6)
**Nationality, n (%)**^b^	
Swiss	21 (75.0)
German	3 (10.7)
Italian	1 (3.6)
Dual citizenship	3 (10.7)
**Native language, n (%)**^b^	
German	24 (85.6)
Italian	1 (3.6)
Bilingual	3 (10.8)
**Residency, n (%)**^a^	
Switzerland	27 (93.1)
Liechtenstein	2 (6.9)
**Highest Education, n (%)**	
Vocational training	8 (26.7)
University degree	22 (73.3)
**Level of Employment, n (%)**^b^	
Full-time (100%)	12 (42.9)
Part-time (20–90%)	16 (57.1)
**Work Experience, mean (SD), years**	16.2 (8.7)
**Annual Income, n (%), CHF**^c^	
0–14,999	1 (4.0)
15, 000–39, 999	2 (8.0)
40, 000–49, 999	6 (24.0)
50, 000–74, 999	6 (24.0)
75, 000 +	8 (32.0)
Do not wish to provide information	2 (8.0)

*Abbreviations*: *SD* Standard deviation, *CHF* Swiss Francs

^a^*N* = 29 due to missing data

^b^*N* = 28 due to missing data

^c^*N* = 25 due to missing data

### Identified themes

#### Theme 1: midwives’ experience with patients with perinatal mental health issues

The identified themes are presented in Table 4. All 30 interviewees reported having had patients who were screened or diagnosed with a perinatal mental disorder over the course of their careers. Notably, 18 midwives recollected that perinatal mental health was not a major topic in midwifery education, and two acknowledged a focus only on postpartum depression.

*«I remember that it was said that this can occur and we learned the differences between “baby blues” and depression. I remember that how to react in these cases and what one is supposed to do was not a big topic» – Midwife 29*

**Table 4:** Interview themes and subthemes

Themes	Subthemes
Midwives’ experience with patients with perinatal mental health issues	
Role of healthcare workers in women’s utilisation of internet-based interventions during the perinatal period	
Overall perception of internet-based interventions	Advantages and expectations
	Disadvantages and concerns
Recommending internet-based interventions to women in the perinatal period	Overall view on internet-based interventions
	Target group

Four participants mentioned that they had personally experienced perinatal mental health issues, of whom three were sceptical about the effectiveness of internet-based interventions and one did not approve of them at all. These participants mostly believed that internet-based interventions are lacking in terms of personal contact. Moreover, the only participant with a negative perception of internet-based interventions stated that perinatal mental health was not a major topic during her midwifery education. The other midwives, who stated that it was an important topic, had an overall positive view of internet-based interventions.

#### Theme 2: role of healthcare workers in women’s utilisation of internet-based interventions in the perinatal period

Midwives and medical doctors such as general practitioners (GPs), gynaecologists or paediatricians were seen by almost all interviewees (*N* = 28) as key figures in the utilisation of internet-based interventions by women with perinatal depression and anxiety. Notably, a third of participants would be willing to take courses on the content and functioning of internet-based interventions in order to increase their knowledge and better support their patients. Of these, 12 also stated that other healthcare professionals should be educated about internet-based interventions and specific mental health online programs (e.g. functioning, content, methodology).

*«I think that all specialists need to take courses on internet-based interventions or information should be given as precisely as possible. Also, workshops or seminars in which one can exactly see what is done in these interventions. » – Midwife 16*

Participants also made suggestions regarding what a successful internet-based intervention would entail, which included involving the patient’s partner (*N* = 7) or baby (*N* = 1), user-friendliness (*N* = 12), interactivity (*N* = 1), availability in different languages (*N* = 1) or availability as a mobile app (*N* = 1). Other suggestions included a mandatory initial face-to-face meeting between patient and psychotherapist (*N* = 1), weekly written communication (*N* = 1) or continued telephone support (*N* = 9).

#### Theme 3: overall perception of internet-based interventions

Twenty-five participants identified internet-based interventions as a valuable intervention strategy, which could be used by the perinatal population. Although four midwives expressed scepticism about the effectiveness of internet-based interventions, only one stated that internet-based interventions are not an acceptable type of intervention and would do more harm than good. Considering these views, the overall perception of internet-based interventions for perinatal mental disorders could be divided into two subthemes: i) advantages and expectations, and ii) disadvantages and concerns.

Several participants mentioned that the main advantages of internet-based interventions are their lower cost (*N* = 14), easy and instant access (*N* = 19), time efficiency (*N* = 12) and anonymity (*N* = 17).

*«They [patients] don’t have to tell anybody that they are taking part* [*in internet-based interventions*]*, also a little anonymity... keep it undercover. » – Midwife 17*

Two thirds of the interviewees viewed internet-based interventions as a low-threshold solution, which might provide help sooner to those in need or facilitate access to psychotherapy. While nine of the interviewees believed that internet-based interventions may help relieve healthcare workers, one expressed that health professionals might feel threatened by the use of internet-based interventions in this population. Finally, four midwives stated that internet-based interventions are useful self-help tools that might encourage women’s self-efficacy and teach them new coping strategies.

On the other hand, participants also identified disadvantages and had concerns regarding internet-based interventions, including neglecting one’s (other) children while taking part in the intervention (*N* = 2), privacy and data protection issues (*N* = 7), potential financial costs (*N* = 3), and high dropout rates and low adherence (*N* = 11). A third of the interviewees also mentioned that internet-based interventions may lead women in the perinatal period to become (more) isolated because the intervention can be done at home.

*«Continued isolation…women will isolate themselves even more. They do it [internet-based interventions] at home or alone. They do not get out. » - Midwife 1*

Additionally, eight participants mentioned that women with perinatal depression or anxiety could easily fake or pretend to be continuously engaged in an internet-based intervention. Five midwives stated that internet-based interventions should focus on aspects involving the entire family system, while two saw the anonymity and flexibility of internet-based interventions as a disadvantage, as patients could postpone using the intervention indefinitely.

#### Theme 4: recommending internet-based interventions to women in the perinatal period

All except for four participants would actively recommend an internet-based intervention to their clients. Yet, one midwife was opposed to recommending any digital tools to women in the perinatal period, and six would prefer to test any online intervention before recommending it.

*«Whether I recommend it [internet-based intervention] would depend on the feasibility of the intervention. How I see it myself, if I think that’s a good thing. When I recommend something like this, I have to be able to “sell” it and have to stand by it. I also sell this as a health professional. That’s why it has to make sense to me. I don’t recommend just anything. » – Midwife 19*

As above, within this theme we identified two main factors influencing midwives’ recommendation: i) overall view on internet-based interventions and ii) target group. A negative view on the effectiveness of internet-based interventions led to not recommending them to patients. However, participants who were sceptical about the effectiveness of internet-based interventions would nevertheless recommend them. Interestingly, participants defined a target group to whom they would recommend an internet-based intervention: A third of the interviewees believed that internet-based interventions were most appropriate for women who are self-aware and intrinsically motivated to do therapy, whereas a fifth believed that internet-based interventions were only appropriate for women who are well-educated or young.

*«Young expectant mothers, I think that’s really about society’s progress. » - Midwife 13*

Although five participants stated that parity would encourage the use of internet-based interventions and influence who could benefit from them, none believed that this was true for women who are primiparous. Moreover, a fifth of the interviewees considered internet-based interventions to be suitable for women from non-Western cultures who lack proficiency in the German language.

*«It would be easier to translate an internet-based intervention, so it might also be a good thing for people with different native languages. » – Midwife 8*

On the other hand, half of the participants expressed concern that women from different cultural backgrounds or with insufficient language and writing skills would have difficulties using internet-based interventions.

*«Perhaps I would not recommend it [*internet-based intervention*] for an immigrant who does not speak enough German. I can imagine that she cannot benefit from it because the language barrier is simply there. » - Midwife 5*

Furthermore, 17 participants expressed that internet-based interventions were not suitable for women with severe mental health disorders, such as severe depression with suicidality or psychosis, and 12 stated that women with a lack of familial or spousal support or with low computer literacy would not benefit from internet-based interventions. Only two midwives stated that they would not recommend internet-based interventions to women who were affected by either traumatic experiences related to computers or an online addiction.

### Quantitative results

An exact Mann-Whitney U test revealed that midwives who reported that healthcare professionals (including themselves) working with the perinatal population should be educated about how internet-based interventions function, and midwives who would be willing to take instructional courses, had a significantly higher percentage level of employment (*U* = 48.500, *p* = .045; *r* = 0.393). Furthermore, we found that participants who made suggestions about internet-based interventions were significantly older than those who did not make any suggestions (*U* = 49.000, *p* = .024; *r* = 0.417). No other significant associations were found between interviewees (*p* > .05).

## Discussion

In this study, we aimed to explore midwives’ views and opinions on the acceptability of internet-based interventions for the treatment of perinatal depression and anxiety symptoms. Our main findings revealed that 97% of the participants perceived internet-based interventions as an acceptable intervention tool entailing several advantages, and would recommend its use to a subgroup of patients. A third of the interviewees would be willing to take courses on the functioning and content of internet-based interventions in order to increase their knowledge and better support their patients. Finally, 20 participants suggested features which could be relevant to develop more acceptable and feasible internet-based interventions in the future.

According to our findings, 25 interviewees have a positive perception of internet-based interventions, believing them to be effective and suitable for use by affected persons. This corresponds to previous studies examining health professionals’ perceptions of internet-based interventions for the postpartum period [59] and for parents of young children with mental health problems [75]. By contrast, with respect to online psychotherapeutic interventions for diagnosed depression, Montero-Marin and colleagues suggested that primary health care personnel might show resistance to patients’ use of internet-based interventions [76].

The advantages and disadvantages identified in the present study were in line with previous literature. For instance, Ashford and colleagues [59] described that health visitors stated that internet-based interventions offer anonymity and flexibility while being a suitable self-help tool for women with postpartum anxiety symptoms. Factors such as cost-effectiveness, convenience, and easy access [35] but also high dropout rates, isolation, faking and privacy concerns were reported as part of internet-based interventions [77, 78]. High dropout rates are an unresolved issue of internet-based therapy programs, [41, 77, 79, 80] but also occur in face-to-face treatments [81]. However, it has been shown that some level of therapist guidance within online interventions may increase adherence, reduce isolation and decrease dropouts [82–84].

Twenty-six midwives in the present study would recommend internet-based interventions to their patients, which is in contrast to a survey involving several countries, including the US, which reported that almost 50% of midwives did not feel confident in referring women in the perinatal period to internet resources [85]. However, half of the present participants mentioned that internet-based interventions were not suitable for women from a different cultural background or who were unfamiliar with the local language, and would also not recommend such programs to women with psychosocial issues such as lack of spousal support. Evidence suggests that women from different cultural backgrounds and women with psychosocial stressors may be at greater risk of severe perinatal mental health problems such as postpartum depression [86–89]. Future studies on technology-based interventions for perinatal depression and anxiety symptoms, addressing cultural and language barriers, limited accessibility to the internet and low computer literacy, are warranted.

Even though 10% of the midwives in our study were sceptical regarding the effectiveness of internet-based interventions, the fact that they would nevertheless recommend them suggests that their commitment to provide patients with all information about the perinatal period, including evidence-based treatment options, may be more relevant than their own personal beliefs [53]. Similarly, previous studies showed that practitioners’ attitudes towards internet-based interventions addressing mild to moderate health problems can range from scepticism to positivity [90, 91]. In addition, 29 participants in the present study defined a target group. For instance, half of the interviewees reported that women with a migration background would not benefit from internet-based interventions, whereas a fifth believed that this method could be suitable for women with various different backgrounds. Evidence indicates that women in the perinatal period who do not understand the local language or have a different cultural background may refrain from using internet-based interventions [54, 55]. Moreover, 17 of our participants would not recommend internet-based interventions to patients with severe mental disorders, which concurs with the recommendations of the National Institute for Health and Care Excellence (NICE) guidelines [92] and with findings on the perceptions of health practitioners [75]. However, none of the interviewees suggested that women should be screened before recommending an internet-based intervention. Comparable to other studies [59, 93, 94], our participants also reported that internet-based interventions would be most suitable for women with higher education, increased self-awareness, younger age, and subclinical levels of depression.

A third of our participants would be willing to educate themselves about the functioning and content of internet-based interventions in order to increase their knowledge and better support their patients. This is a relevant finding, as it is critical to evaluate the quality of information that is provided within online resources. Surprisingly, however, a survey of midwives from around the world revealed that approximately 20% of the sample were unsure about or disagreed with incorporating internet use and resources as part of midwifery training [85]. This is concerning in light of previous findings that a successful and consistent utilisation and recommendation of internet-based interventions requires awareness, acceptance, and training from key healthcare workers [95–97]. Appropriate training as well as information courses could potentially help midwives to gain more knowledge and awareness about the effectiveness of internet-based interventions for women with mild to moderate depression and anxiety symptoms.

Finally, 20 participants made suggestions to improve internet-based interventions. The interview guide did not contain any specific questions covering potential suggestions on how to improve internet-based interventions, but most midwives put forward ideas on how they imagined a successful internet-based intervention should look. Interestingly, participants who made suggestions regarding the content of internet-based interventions were significantly older than those who did not. Future studies may want to examine whether healthcare workers’ increased age is associated with more experience in working with perinatal women.

As a limitation of the present study, participants only received a short text about internet-based interventions, which might have been insufficient for those with no prior knowledge of such interventions. Nevertheless, the participants were encouraged to ask questions at any time. The strengths of the study include the use of framework analysis, which focuses on data management, improves transparency about interpretations [98, 99], and allows for data comparison within and between participants [68]. Moreover, we followed the COREQ [67] guidelines in reporting qualitative data and used Krippendorff’s alpha to demonstrate reliable and coherent coding between different raters [100]. The study encompassed almost twice as many participants as in other similar studies [59, 101], and the sample size can be regarded as relatively large [102, 103]. Finally, the study is one of the few to explore the views and experiences of healthcare professionals regarding the use of internet-based interventions for perinatal depression and anxiety symptoms, thus providing a valuable contribution to the literature [59, 104]. While our findings cannot be transferred beyond the German-speaking Swiss/Liechtenstein context, the sample was diverse in terms of work experience and location.

## Conclusions

In sum, our findings suggest that the interviewed midwives have a positive perception of internet-based interventions, despite identifying disadvantages and having concerns about their use. These findings are encouraging, and contribute to the continued efforts to develop internet-based mental health interventions as a way to support women screened or diagnosed with mild to moderate depression and/or anxiety symptoms during pregnancy and postpartum. Furthermore, 26 of 30 participants would recommend internet-based interventions to a subgroup of their patients, see themselves and medical doctors as key figures in terms of recommending internet-based interventions, and would be willing to learn more about how they function. Healthcare professionals who have contact with women during pregnancy and postpartum play an important role in evaluating and directing them to high-quality online resources. To achieve this, their knowledge and skills need to be developed such that they can be effective and competent in supporting patients. Future studies should explore other relevant factors for the acceptability of internet-based interventions among this population, such as openness to new treatments. Moreover, it would be useful to examine attitudes and perceptions of a range of healthcare workers, such as primary care doctors or obstetricians, regarding the use of internet-based interventions for mild to moderate depression and anxiety symptoms. Our findings have implications not only for midwives’ practice, but also for other maternity care professionals.

## Data Availability

The datasets used and analysed during the current study are available from the corresponding author on reasonable request.

## Abbreviations

FSP: Federation of Swiss Psychologists
COREQ: Consolidated Criteria for Reporting Qualitative Research Checklist
GPs: General practitioners
NICE: National Institute for Health and Care Excellence
